# Miller Fisher Syndrome Presenting Without Areflexia, Ophthalmoplegia, and Albuminocytological Dissociation: A Case Report

**DOI:** 10.7759/cureus.23371

**Published:** 2022-03-21

**Authors:** Mohammad R Ghani, Muhammad Ismail Khalid Yousaf, Kelly Van Bussum, Ping Shi, Rolando M Cordoves Feria, Martin Brown

**Affiliations:** 1 Neurology, University of Louisville School of Medicine, Louisville, USA; 2 Neurology, Jewish Hospital, UofL Health, Louisville, USA

**Keywords:** guillain-barré syndrome (gbs), albuminocytological dissociation, neuromuscular diseases, intravenous immunoglobulins (ivig), miller fisher syndrome (mfs)

## Abstract

Miller Fisher syndrome (MFS) is a rare variant of Guillain-Barré syndrome (GBS) with a prevalence of one to two people per million each year. Viral and/or bacterial infection often precedes the classic triad of areflexia, ophthalmoplegia, and ataxia. Bulbar involvement is uncommon but can lead to extensive workup to rule out stroke, myasthenia gravis (MG), and other neuromuscular disorders. We present a case of a 32-year-old healthy male with a past medical history of Lyme disease as a teenager and sore throat two weeks prior. He presented to the hospital with rapidly ascending paresthesias in bilateral upper and lower extremities, urinary incontinence, and mild slurred speech. Exam on presentation revealed mild dysmetria in bilateral upper and lower limbs. The remainder of the exam was negative. Neuroradiological imaging, including magnetic resonance imaging (MRI) with and without contrast of the brain and the cervical and lumbar spine, did not show any acute process or abnormal enhancement. Lumbar puncture revealed cerebrospinal fluid (CSF) with normal protein and cell count, and hence no albuminocytological dissociation (ACD). Immunoserology was positive for Epstein-Barr virus (EBV) immunoglobulin G (IgG) but negative for immunoglobulin M (IgM). Despite the absent ACD, areflexia, and no third, fourth, and sixth cranial nerve deficits, there was high suspicion for GBS due to acutely rapid ascending paresthesia, mild dysarthria, and mild ataxia. The patient was started on intravenous immunoglobulin (IVIG) 2 mg/kg divided into five days within 24 hours of admission. The patient developed areflexia in all limbs on the second day of admission and complained of double vision. On the third day of admission, the patient's negative respiratory force (NIF) declined to −23, and he was intubated for airway protection. Our patient completed five days of IVIG. Positive anti-GQ1b antibodies further supported the diagnosis of MFS. After a seven-day ICU stay and 20 days of aggressive inpatient rehabilitation, the patient could do most of the activities of daily living independently. After six weeks, he was back to his normal baseline and restarted his job.

## Introduction

Dr. Charles Miller Fisher first described Miller Fisher syndrome (MFS) with three defining features: ophthalmoplegia, ataxia, and areflexia [1]. The incidence of Guillain-Barré syndrome (GBS) is one to two per 100,000 individuals, and MFS only accounts for 5-10% of those cases [2]. MFS affects twice as many males as it does women [2]. People of all ages can be affected, with the median age of onset in the fifth decade [3]. Diplopia (78%) and ataxia (48%) are prevalent symptoms of MFS, while ophthalmoplegia accounts for almost 34% [2]. Leg dysesthesia, blepharoptosis, facial, bulbar, and pupillary palsies, moderate (grade 4) motor weakness, and micturition disruption are among the less common symptoms [3]. These symptoms typically develop rapidly over a few days, preceded by viral or bacterial infection. MFS diagnosis is a clinical one. Cerebrospinal fluid (CSF) studies, electrodiagnostic studies, and serologic analysis help confirm the diagnosis [4]. Anti-GQ1b antibodies are present in more than 80% of MFS patients [5,6]. The prognosis of MFS patients is generally good, with baseline average recovery within two to six weeks from the onset of symptoms. Treatment for MFS is mainly adequate supportive care. In moderate to severe cases, intravenous immunoglobulin (IVIG) or plasmapheresis is indicated [7]. It is essential to diagnose and treat MFS patients emergently as respiratory depression can lead to intubation and prolonged hospitalization.

## Case presentation

A 32-year-old right-handed Caucasian male with a past medical history of Lyme disease as a teenager, otherwise active and at good baseline health, presented to the emergency department after waking up with rapidly ascending paresthesias in bilateral upper and lower extremities, urinary incontinence, and mild slurred speech noticed by his wife. In addition, he reported an improved sore throat, which had been symptomatic for almost two weeks prior to presentation. Besides sulindac for sore throat, he did not take any medications. His wife also reported a similar sore throat three weeks back. His family history was positive for multiple sclerosis in his mother. He denied any history of smoking, illicit drug, or alcohol abuse.

On initial examination, the patient was awake, alert, and oriented to person, place, time, and events. His vital signs were within normal limits. His abnormal findings included mild dysarthria, mild dysmetria observed with finger to nose and heel to shin testing bilaterally, and decreased temperature and vibration sensations in all extremities bilaterally and distally. He denied visual field deficits, and extraocular movements were intact without nystagmus. His strength was 5/5 in all limbs and intact gross and light touch with pain localization. His reflexes were symmetric and 2+ in triceps, biceps, brachioradialis, and patellar, and 1+ in Achilles bilaterally. The patient denied diplopia, ptosis, vision loss, dysphagia, sialorrhea, shortness of air, loss of consciousness, and dizziness.

The patient's complete blood count, comprehensive metabolic panel, thyroid panel, urinalysis, C-reactive protein, and erythrocyte sedimentation rate (ESR) were within normal limits, and the urine toxicology screen was negative. Immunoserology was positive for Epstein-Barr virus (EBV) immunoglobulin G (IgG) but negative for immunoglobulin M (IgM). Coronavirus disease 2019 (COVID-19), human immunodeficiency virus (HIV), and rapid plasma reagin (RPR) tests were also negative. Vitamin B12 was low at 189, and he was started on subcutaneous vitamin B12 replacement.

Computed tomography (CT) of the brain was negative for any acute process. Magnetic resonance imaging (MRI) of the brain and the cervical and lumbar spine, all without and with contrast, were ordered to look for any inflammatory pathology such as demyelinating lesions or peripheral nerve root enhancements. Both cervical and lumbar spine imaging revealed mild bone degenerative changes, but no cord signal change, demyelinating lesions, or nerve root enhancement was visualized. MRI of the brain was also unremarkable. Lumbar puncture showed normal protein and cell count with no albuminocytological dissociation (ACD). Although on examination, the patient did not have areflexia, ophthalmoplegia, limb weakness, dysphagia, and ACD on lumbar puncture, there was a high suspicion of GBS due to acute rapidly ascending paresthesias, dysarthria, and ataxia in the context of a recent sore throat. The patient was started on IVIG with a dose of 2 gm/kg divided into five days. His negative inspiratory force (NIF) was checked every four hours.

On day two of hospitalization, the patient reported diplopia and developed areflexia in the lower limbs. On day three, he had sudden onset respiratory distress and was emergently transferred to the intensive care unit (ICU). The patient went into respiratory distress with a NIF of −23 leading to intubation for airway protection. He completed five days of IVIG. The patient also received gabapentin, which was later discontinued due to new-onset agitation. He self-extubated on hospital day six and remained extubated. He was transferred out of the ICU to an intermediate floor on hospital day seven. After extubation, a detailed neurologic exam revealed a vertical gaze palsy and variable restriction of extraocular movements in all directions along with diffuse areflexia and ataxia of bilateral upper and lower extremities.

The patient reported mild improvement in speech, ophthalmoplegia, and ataxia after working with speech and occupational therapy in the hospital. He needed moderate aid with ambulation due to ataxia but could feed himself and stand on his feet. However, he continued to report diplopia as his most significant obstacle in physical therapy. Physiatry was consulted and recommended acute inpatient rehabilitation upon discharge from the hospital given his persistent functional impairment. Once at inpatient rehabilitation, he improved with therapy and was discharged home after one week.

The patient's serum antibody testing was positive for anti-GQ1b IgG, which along with other findings during the hospital course, confirmed the diagnosis of MFS.

## Discussion

The common variants of GBS include acute inflammatory demyelinating polyneuropathy (AIDP), acute motor axonal neuropathy (AMAN), Bickerstaff brainstem encephalitis (BBE), pharyngeal-cervical-brachial (PCB) weakness, and MFS. All these variants have in common an underlying autoimmune inflammatory process with antibodies to gangliosides on the axolemma targeting macrophages to invade the axon at the node of Ranvier [8,9].

In 56-76% of patients with GBS, these clinical signs are preceded by upper respiratory or gastrointestinal tract illnesses. *Campylobacter jejuni* and *Haemophilus influenzae* are the most frequent pathogens. Other common pathogens associated are *Mycoplasma pneumoniae* and *Cytomegalovirus* (CMV) [9]. EBV is less frequently reported as the primary pathogen preceding MFS.

GBS usually develops quickly, with neurologic symptoms appearing 8-10 days (range 1-30 days) after the antecedent illness [2]. Following the onset of neurologic symptoms, the condition advances until it reaches a clinical nadir at around a week (range of 2-21 days) [2]. MFS is diagnosed by clinical history, cardinal symptoms, abnormal nerve conduction studies, and ACD in the CSF.

Anti-GQ1b ganglioside antibodies in MFS were first described by Chiba et al. [10]. Antibodies against gangliosides play an essential role in diagnosing MFS and other GBS variants and express distinct clinical characteristics. Gangliosides (GM1, GD1b, GD1a, and GQ1b) are neuronal components abundantly found in different central and peripheral nervous tissues. They play an essential role in the development and repair of neuronal cells [11]. Anti-GQ1b antibodies limit acetylcholine release from motor nerve terminals by binding to GQ1b. GQ1b antigens are found more in the cranial nerves three, four, and six and muscle spindles in the limbs [12]. These antibodies are present in almost 80-95% of patients with MFS, correlating with disease activity and facilitating MFS diagnosis [10].

Although anti-GQ1b antibodies are not specific to MFS, they aid in serological confirmation, allowing for a more precise diagnosis in the context of cofounding symptoms. It is crucial to cautiously differentiate other disorders with positive anti-GQ1b antibodies, including BBE and PCB weakness. All three conditions have in common the underlying pathogenesis with GQ1b autoimmune reactivity and antecedent prodromal infection-causing demyelinating peripheral neuropathy [13].

In BBE, GQ1b antibodies are seen mainly in the brainstem. Therefore, other structures such as cranial nerves, pyramidal tracts, and brainstem reticular activating systems can also be involved. BBE is clinically characterized by encephalopathy with ophthalmoplegia, ataxia, and hyperreflexia. On imaging, some patients show hypodensity changes in the brainstem (Table 1) [14,15].

**Table 1:** Clinical characteristics of GQ1b+ syndromes.

Diseases/characteristics	Miller Fisher syndrome (MFS)	Bickerstaff brainstem encephalitis (BBE)	Pharyngeal-cervical-brachial (PCB) weakness
Common signs and symptoms	Ophthalmoplegia, ataxia, and areflexia	Encephalopathy with ophthalmoplegia and ataxia	Acute weakness of the oropharyngeal, neck, and shoulder muscles with swallowing dysfunction

The clinical features of PCB weakness are rapid weakness in the oropharyngeal and cervicobrachial area with areflexia in the upper limbs in the absence of ophthalmoplegia or leg weakness. The symptoms of PCB are similar to MFS and BBE (Table 1), preceded by upper respiratory or gastrointestinal tract infection symptoms. Almost half of the patients with PCB have IgG anti-GT1a antibodies, which often cross-react with GQ1b. In MFS, most antibodies found are against the GQ1b antigen, which can cross-react with GT1a [16].

Our patient's early presentation after an upper respiratory ailment without ophthalmoplegia and areflexia but rapidly developing ascending paresthesia and positive EBV IgG warranted a GBS workup. Many different viruses and bacteria have been associated with the development of GBS. Not many cases of adult patients to our knowledge are in the literature that has associated EBV with MFS to date, with undetermined incidence. Chang et al. describe EBV as a possible causative agent for MFS, speculating the underlying molecular structure of the microbial lipopolysaccharide (LPS) of EBV has molecular mimicry with GQ1b, which triggers anti-GQ1b IgG attack against autogenous neurons, which results in peripheral nerve damage [17]. Similar to the case by Chang et al., our patient could have had the appearance of positive GQ1b antibodies related to the EBV infection.

The management approach for our patient with initial IVIG displayed a rapid decline followed by improvement of his condition, which is related to a good prognosis. Our findings are confirmed by prior research that found a considerable reduction in hospital stay and a shorter length of mechanical breathing to compensate for the expense of plasmapheresis and improved secondary outcomes [18]. Van der Meche and Schmitz described patients treated with IVIG are at least as effective as plasma exchange and maybe superior [19]. Patients who got IVIG therapy, on the other hand, appeared to improve more than those who had plasmapheresis [17].

## Conclusions

In conclusion, diagnosing early-onset MFS can be challenging at times. Knowing its classical presentation and variants helps in early diagnosis and management. ACD in the spinal fluid examination can be absent in the first week of presentation, which does not exclude the possibility of demyelinating polyneuropathy. Clinical worsening can be seen even after the initiation of therapy. Overall, IVIG is generally better tolerated and more accessible than plasmapheresis, as evident in our patient.
